# Biochar application increases sorption of nitrification inhibitor 3,4-dimethylpyrazole phosphate in soil

**DOI:** 10.1007/s11356-018-1658-2

**Published:** 2018-03-08

**Authors:** Katharina M. Keiblinger, Franz Zehetner, Axel Mentler, Sophie Zechmeister-Boltenstern

**Affiliations:** Institute of Soil Research, Department of Forest and Soil Sciences, University of Natural Resources and Life Sciences Vienna (BOKU), Peter Jordan Strasse 82, A-1190 Vienna, Austria

**Keywords:** Nitrification inhibitor, Biochar, Nitrogen cycle, DMPP, Hydrophobicity

## Abstract

Biochar (BC) application to soils is of growing interest as a strategy to improve soil fertility and mitigate climate change. However, BC-induced alterations in the soil N cycle are currently under debate. BC has recently been shown to accelerate the emissions of N_2_O via the biotic ammonium oxidation pathway, which results in lower nitrogen use efficiency and environmentally harmful losses of NO_3_ and/ or N_2_O. To avoid these potential losses, the use of nitrification inhibitor (NI) could provide a useful mitigation strategy for BC-amended agricultural fields. Here, we tested the sorption behavior of a model NI, the synthetic 3,4-dimethylpyrazole phosphate (DMPP) on 15-month-aged soil-BC mixtures. We saw that BC additions increased DMPP sorption to varying extents depending on BC feedstock type and pyrolysis temperature. The highest sorption was found for BC pyrolyzed at a lower temperature. BC effects on soil physico-chemical characteristics (i.e., hydrophobicity) seem to be important factors.

## Introduction

Biochar (BC) is a carbonaceous material produced by pyrolysis of biomass. Its application to soil is a potential strategy to improve soil quality in an efficient and sustained manner (Lehmann and Joseph 2009). Moreover, it constitutes an opportunity for climate change mitigation by C-sequestration in soils due to its highly chemically recalcitrant nature and its stability against decomposition (Lehmann and Joseph 2009). Also, BCs added to soils have the potential to reduce NO_3_ losses and N_2_O emissions (i.e., Van Zwieten et al. 2015). N_2_O is produced via a large set of reactions, i.e., nitrification and denitrification pathways (Butterbach-Bahl et al. 2013), both largely responsible for N losses in agriculture (Subbarao et al. 2013). The reduction of N_2_O losses with BC application is most likely attributed to the pathway of total denitrification to N_2_ (Cayuela et al. 2013). However, the positive effect of BC application on soil N_2_O emissions is currently under debate (Sánchez-García et al. 2014; Cayuela et al. 2013). For example, a recent study reported increased N_2_O losses via the biotic processes of ammonia oxidation and nitrifier-denitrification (Sánchez-García et al. 2014). This pathway of N_2_O losses is supported by higher abundances of ammonia-oxidizing communities together with accelerated gross nitrification rates in BC-amended agricultural soils (Prommer et al. 2014). The BCs’ liming effect may increase the pH of acidic soils, rendering conditions more optimal for soil nitrifiers (Prosser and Nicol 2012).

One way to overcome high N losses via nitrification are nitrification inhibitors (NIs), thereby improving nitrogen use efficiency in agricultural soils and reducing climate-relevant N_2_O emissions (Friedl et al. 2017; Yang et al. 2016). They facilitate the retention of soil N in the form of NH_4_ by inhibiting the ammonium monooxygenase (AMO) through random binding to the membrane-bound enzyme. AMO shows a broad substrate range (Marsden et al. 2016), and inhibitors compete with NH_4_ for the active site of this enzyme, thereby delaying the first and rate-limiting step of nitrification (Zerulla et al. 2001). To avoid enhanced nitrification and associated N losses, the use of synthetic NI could provide a valuable strategy for BC-amended agricultural fields. Yet, little is known about the effect of BC on the sorption of NI in soil beside the theory that accelerated net nitrification in BC-amended ecosystems may be due to the removal of natural NI such as polyphenols and tannins (Clough et al. 2013). The sorption/desorption of organic molecules in soil is mainly influenced by the organic matter content and physico-chemical properties that can be strongly altered through BC addition (i.e., polarity and charge properties of adsorbates and soil particles, and microbial reactions). As BC amendment can add new binding sites and strongly alter soil physico-chemical characteristics (i.e., pH, hydrophobicity) (Rechberger et al. 2017; Kumari et al. 2014), it may affect the sorption behavior of NI in the soil environment. Among the most widely used commercial NIs, 3,4-dimethylpyrazole phosphate (DMPP) is highly efficient at reducing N losses at low application rates (Benckiser et al. 2013), shows minor eco-toxicological side effects on plants (Yang et al. 2016), and is less mobile than the inhibitor dicyandiamide (DCD), due to sorption (Wissemeier et al. 2001). Hence, this experiment aimed at assessing the effect of BC application on the sorption of the synthetic NI, DMPP as a model NI, in soil-BC mixtures. More specifically, we investigate the effect of BC quality on DMPP sorption.

## Methods

Soil-BC mixtures were obtained from a greenhouse pot experiment, described in Kloss et al. (2014). Briefly, an acidic (pH 5.4) planosol with sandy loam texture (10.7% clay, 19.6% silt, and 69.8% sand), C_org_ 1.6%, electrical conductivity (EC) 41.2 μS cm^−1^, and CEC 75.1 mmol_c_ kg^−1^ (Kloss et al. 2014) was incubated for 15 months with different BC types. The biochars had been produced from three different feedstocks: woodchips (WC), wheat straw (WS), both slowly pyrolyzed at 525 °C, and vineyard pruning (VP) pyrolyzed at different temperatures, i.e., 400 °C (T1) and 525 °C (T2), respectively. Detailed description of the BC pyrolysis is provided in Kloss et al. (2012). The BCs were applied at 3 wt% (Kloss et al. 2014), with the WC-derived BC also applied at 1 wt%.

**Fig. 1:**
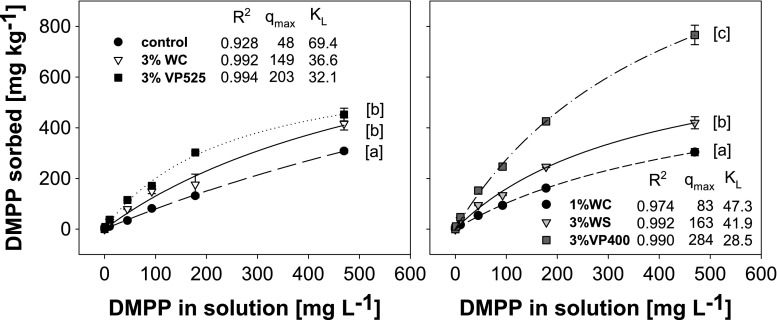
Langmuir isotherms for DMPP sorbed to control soil and soil-BC mixtures, with BC derived from woodchips (WC), vineyard pruning (VP), and wheat straw (WS), applied at a concentration of 1 and 3% and varying pyrolysis temperature (T1, 400 °C; T2, 525 °C). The Langmuir equation (Eq. 1) was used to fit the data (0.928 < *R*^2^ < 0.994). Different letters indicate statistical significance between treatments at the highest concentration using ANOVA with Duncan’s multiple range test (*p* < 0.05, *n* = 3). The parameters for the Langmuir isotherm *q*_max_ (mg kg^−1^) and *K*_L_ (L g^−1^) are shown (*n* = 3). Error bars indicate standard deviation

We used 3,4-dimethylpyrazole phosphate (DMPP) to evaluate the equilibrium state sorption characteristics in six different soil-BC mixtures (control soil, WC1%, WC3%, WS3%, VP3%T1, VP3%T2). DMPP in liquid solution with 72% *w*/*v* was used as received from BASF Ag, Ludwigshafen, Germany, without further purification. DMPP was added in a 1:10-*w*/*v* ratio to 1 g well-homogenized (sieved < 2 mm) soil-BC mixture at six concentrations (1, 10, 50, 100, 200, 500 mg L^−1^) in three replicate 15-mL centrifuge tubes. In order to reduce some of the factors that strongly influence sorption/desorption of organic molecules in soil, the following procedures were performed. The solutions were prepared in phosphate buffer (pH 7) to hold the charge properties of the adsorbate (DMPP) constant among all treatments. In addition, each solution contained 3 mM NaN_3_ to eliminate microbial activity. Solutions were shaken at room temperature for 24 h to ensure equilibrium conditions. Subsequently, the tubes were centrifuged for 15 min at 3000*g*. The original solution and the supernatants were then filtered through 0.45-μm membrane filters before isocratic HPLC analysis using 3,4-dimethylpyrazole (DMP, Sigma Aldrich) as a standard (range 0.39 up to 100 mg L^-1^ in serial dilutions). Standards and samples were separated on a reversed-phase C18 column and measured with a UV detector at 224 nm according to German Standards (OENorm-EN16328 2013). The amount of sorbed DMPP vs. the DMPP concentration in solution was plotted in Fig. 1, and sorption isotherms were fitted using the Langmuir equation:

1 $$ {q}_{\mathrm{e}=}{q}_{\mathrm{max}}\frac{K_{\mathrm{L}}\times {c}_{\mathrm{e}\mathrm{q}}}{1+{K}_{\mathrm{L}}\times {c}_{\mathrm{e}\mathrm{q}}}, $$

where *q*_e_ is the solid phase concentration adsorbed, *c*_eq_ is the equilibrium liquid phase concentration, *q*_max_ is the maximum load of sorption corresponding to complete coverage, and *K*_L_ the ratio of sorption and desorption rates (Chong and Volesky 1995). The Langmuir isotherm is suitable for physical monolayer sorption in the lower concentration range (Meghea et al. 1998). In our sorption experiment, also the electrical conductivity (EC) was measured in the supernatants after 24-h shaking in three replicates (Table 1).

**Table 1:** Basic characteristics of biochars and soil-biochar mixtures (according to Kloss et al. (2014) and Keiblinger et al. (2015))

	SSA, (m^2^ g^−1^)	Contact angle, (°)	Ash content, (wt%)	EC, (mS cm^−1^)	OC	pH (CaCl_2_) (1)
Control soil				18.36 ± 0.03	1.6 ± 0.5^a^	5.5 ± 0.05^a^
WC1%	26.4 ± 0.8^d^	13.8 ± 17.5^a^	15.2	17.91 ± 0.18	1.7 ± 0.3^a^	6.5 ± 0.07^c^
WC3%				18.50 ± 0.03	3.5 ± 0.6^ab^	6.7 ± 0.01^d^
WS3%	12.3 ± 1.3^c^	21.3 ± 9.1^a^	28.1	18.38 ± 0.05	4.6 ± 0.9^b^	6.3 ± 0.00^b^
VP3%T1	1.7 ± 0.1^a^	73.8 ± 15.9^c^	4.3	18.41 ± 0.07	3.3 ± 2.0^ab^	6.4 ± 0.00^c^
VP3%T2	4.8 ± 0.3^b^	49.2 ± 17.7^b^	7.7	18.00 ± 0.20	4.3 ± 1.4^b^	6.5 ± 0.02^c^

Biochars derived from woodchips (WC), vineyard pruning (VP), and wheat straw (WS), applied at a concentration of 1 and 3% and varying pyrolysis temperature (T1, 400 °C; T2, 525 °C); values are mean ± standard error. Specific surface area (SSA) and ash content were determined from the initial biochars before application; the organic carbon (OC) was measured after 7-month incubation in the pot experiment (four replicates); the water contact angle (a measure of hydrophobicity) of the BCs after 15 months. The electrical conductivity (EC) was measured from the supernatants; different letters indicate significant differences (*p* < 0.05; one-way ANOVA with Duncan’s multiple range test)

To characterize the sorption behavior in more detail, the sorption constants were related to the physico-chemical properties of the studied BCs and soil-BC mixtures. The specific surface area (SSA) and ash content were determined for the initial BCs before soil application, and organic carbon (OC) and C:N of soil-BC mixtures were measured after 7-month incubation in the pot experiment (four replicates); the values are taken from Kloss et al. (2014). The contact angle or hydrophobicity of the BCs after 15 months was reported earlier by Keiblinger et al. (2015).

Isotherms were fitted to the experimental sorption data using Sigma Plot 12.5. Treatments were compared with ANOVA followed by Duncan’s multiple range test (*p* < 0.05). Correlation analysis between sorption constants and BC physico-chemical characteristics was conducted using Statgraphics Centurion.

## Results and discussion

The pH measured after the sorption procedure ranged from 6.93 to 6.97 for the highest concentration of DMPP. The neutral pH, at which our sorption experiments were buffered, reflects the BCs’ liming effect; indeed, the pH value of the studied planosol was raised from 5.4 into the neutral range through BC addition (Kloss et al. 2014). While under acidic conditions (pH < 4.5), the hydrolysis equilibrium of the molecule DMPP shifts towards the DMP^+^ cation, at higher pH values (> 4.5), and DMPP hydrolyzes in solution and is present in neutral form (Shi et al. 2016). The latter is supported by a plateau observed for the equilibrium adsorption capacity of DMPP between pH 4.5 and 9 (Shi et al. 2016).

In the present experiment, sorption of DMPP decreased in the order: VP3%T1 > VP3%T2 ~ WS3% ~ WC3% > WC1% ~ control soil (Fig. 1). Already at the lowest DMPP concentration (1 mg L^−1^), there was a significant difference (*p* < 0.05) between the treatments; this difference remained similar as DMPP concentrations increased. We found no clear difference in DMPP sorption constants between different BC feedstock types, but lower DMPP sorption for the lower application rate in the soil-BC mixture of WC1% vs. WC3% (Fig. 1). The lowest sorption was observed for control soil and WC1%, which may be due to its low organic C content (OC, Table 1). This was also corroborated by a positive linear relationship between Langmuir constant *q*_max_ and OC of soil-BC mixtures was observed (*r* = 0.81; *p* = 0.05). Significantly greater DMPP sorption was found for the treatment with VP pyrolyzed at 400 °C (T1) vs. 525 °C (T2) (Fig. 1). This could be related to hydrophobic interactions, as hydrophobicity decreases with increasing pyrolysis temperature (Zornoza et al. 2016), and VP3%T1 indeed showed a higher contact angle than VP3%T2 (Table 1). Over all BCs, significant differences in hydrophobicity were observed, even though these had been incubated in soil for 15 months. The increased sorption of DMPP after BC application may be caused by the hydrophobicity of the BC samples (Table 1), as there is a positive linear relationship between *q*_max_ and the BCs’ contact angle (*r* = 0.9796; *p* = 0.0204). At the neutral pH of the present sorption study, the DMPP molecule was uncharged and, thus, it is likely that hydrophobic interactions have controlled DMPP sorption. This assumption is consistent with previous results showing that organic molecules, such as oxytetracycline, are predominantly sorbed through hydrophobic interactions when the molecule is a zwitterion, while at lower pH when the molecule has a net positive charge, cationic exchange interactions are dominant (Kulshrestha et al. 2004). Hydrophobic interactions between BC and DMPP also seem plausible considering that pyrazole methylation increases hydrophobicity (Hartman et al. 2013).

However, with BC aging (the change in properties of BC due to the physical and biogeochemical interaction of BC in soil, Kumari et al. 2014), decreases in BC hydrophobicity have been found (Rechberger et al. 2017), which may reduce their sorption capacity for hydrolyzed, neutral organic molecules over time. The sorption of DMPP to soil-BC mixtures observed in our study likely decreases its availability to microbes, which could reduce its inhibitory effect but also protect it from decay. Further studies are needed to clarify the resulting effect on the soil nitrogen cycle.
